# Neutralization of SARS-CoV-2 lineage B.1.1.7 pseudovirus by BNT162b2 vaccine–elicited human sera

**DOI:** 10.1126/science.abg6105

**Published:** 2021-01-29

**Authors:** Alexander Muik, Ann-Kathrin Wallisch, Bianca Sänger, Kena A. Swanson, Julia Mühl, Wei Chen, Hui Cai, Daniel Maurus, Ritu Sarkar, Özlem Türeci, Philip R. Dormitzer, Uğur Şahin

**Affiliations:** 1 BioNTech, An der Goldgrube 12, 55131 Mainz, Germany.; 2 Pfizer, 401 N. Middletown Rd., Pearl River, NY 10960, USA.; 3 TRON gGmbH – Translational Oncology at the University Medical Center of the Johannes Gutenberg University, Freiligrathstraße 12, 55131 Mainz, Germany.

## Abstract

Recently, a new severe acute respiratory syndrome coronavirus 2 (SARS-CoV-2) lineage called B.1.1.7 (variant of concern: VOC 202012/01), which is reported to spread more efficiently and faster than other strains, emerged in the United Kingdom. This variant has an unusually large number of mutations, with 10 amino acid changes in the spike (S) protein, raising concerns that its recognition by neutralizing antibodies may be affected. In this study, we tested SARS-CoV-2-S pseudoviruses bearing either the Wuhan reference strain or the B.1.1.7 lineage spike protein with sera of 40 participants who were vaccinated in a previously reported trial with the messenger RNA–based COVID-19 vaccine BNT162b2. The immune sera had slightly reduced but overall largely preserved neutralizing titers against the B.1.1.7 lineage pseudovirus. These data indicate that the B.1.1.7 lineage will not escape BNT162b2-mediated protection.

## Vaccine protects against B1.1.7 variant

The severe acute respiratory syndrome coronavirus 2 (SARS-CoV-2) B1.1.7 (VOC 202012/01) variant that emerged in late 2020 in the United Kingdom has many changes in the spike protein gene. Three of these are associated with enhanced infectivity and transmissibility, and there are concerns that B.1.1.7 might compromise the effectiveness of the vaccine. Muik *et al.* compared the neutralization efficacy of sera from 40 subjects immunized with the BioNTech-Pfizer mRNA vaccine BNT162b2 against a pseudovirus bearing the Wuhan reference strain or the lineage B.1.1.7 spike protein (see the Perspective by Altmann *et al.*). Serum was derived from 40 subjects in two age groups 21 days after the booster shot. The vaccine remained effective against B.1.1.7 with a slight but significant decrease in neutralization that was more apparent in participants under 55 years of age. Thus, the vaccine provides a significant “cushion” of protection against this variant.

*Science*, this issue p. 1152; see also p. 1103

## Teaser

Despite the many genetic changes in the B.1.1.7 (VOC 202012/01) 2020 UK variant of SARS-CoV-2, the BioNTech-Pfizer mRNA vaccine remains protective.

In a phase 3 trial conducted in the United States, Argentina, Brazil, South Africa, Germany, and Turkey, the BioNTech-Pfizer mRNA vaccine BNT162b2 was 95% effective in preventing COVID-19 through the data cutoff date of 14 November 2020 (*1*). The severe acute respiratory syndrome coronavirus 2 (SARS-CoV-2) lineage B.1.1.7 (variant of concern: VOC 202012/01) was discovered to have emerged in the United Kingdom in September 2020 (*2*), and it subsequently increased in prevalence, showed enhanced transmissibility, and spread to other countries and continents (*3*). B.1.1.7 has a series of mutations in its spike (S) protein: ΔH69/V70, ΔY144, N501Y, A570D, D614G, P681H, T716I, S982A, and D1118H (H, His; V, Val; Y, Tyr; N, Asn; A, Ala; D, Asp; G, Gly; P, Pro; T, Thr; I, Ile; S, Ser). One of these mutations, N501Y, was of particular concern because it is located in the receptor binding site. The spike with this mutation binds more tightly to its cellular receptor, ACE-2 (*4*), and virus with this mutation has an increased host range that includes mice (*5*). BNT162b2-immune sera neutralized SARS-CoV-2 (USA/WA-1/2020 background strain) with an introduced N501Y mutation as efficiently as they neutralized SARS-CoV-2 without the mutation (*6*). Further, 19 pseudoviruses, each bearing a SARS-CoV-2 S with a different mutation found in circulating virus strains, were also neutralized as efficiently as nonmutant SARS-CoV-2 S–bearing pseudoviruses by BNT162b2-immune sera (*7*). However, it was still unclear whether a virus with the full set of mutations in the lineage B.1.1.7 spike, each of which may potentially interfere with antibody binding, would be neutralized efficiently by BNT162b2-immune sera.

**Fig. 1:**
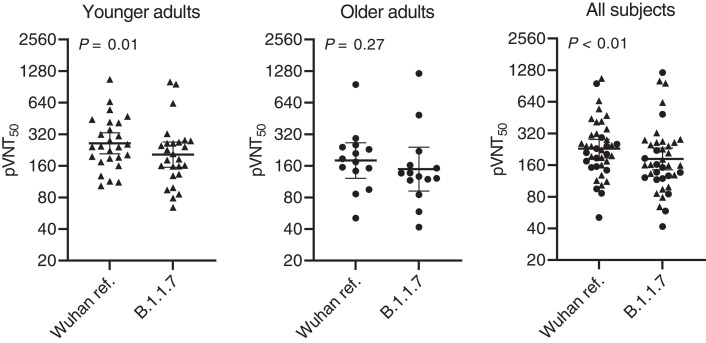
50% pseudovirus neutralization titers (pVNT_50_) of 40 sera from BNT162b2 vaccine recipients against VSV-SARS-CoV-2-S pseudovirus bearing the Wuhan reference strain or lineage B.1.1.7 spike protein. Sera from *n* = 26 younger adults (aged 23 to 55 years; indicated by triangles) and *n* = 14 older adults (aged 57 to 73 years; indicated by circles) drawn at either day 29 or day 43 (7 or 21 days after vaccine dose two) were tested. Statistical significance of the difference between the neutralization of the VSV-SARS-CoV-2-S pseudovirus bearing the Wuhan or lineage B.1.1.7 spike protein was calculated by a Wilcoxon matched-pairs signed rank test. Two-tailed *P* values are reported. GMTs and 95% CIs are indicated.

**Fig. 2:**
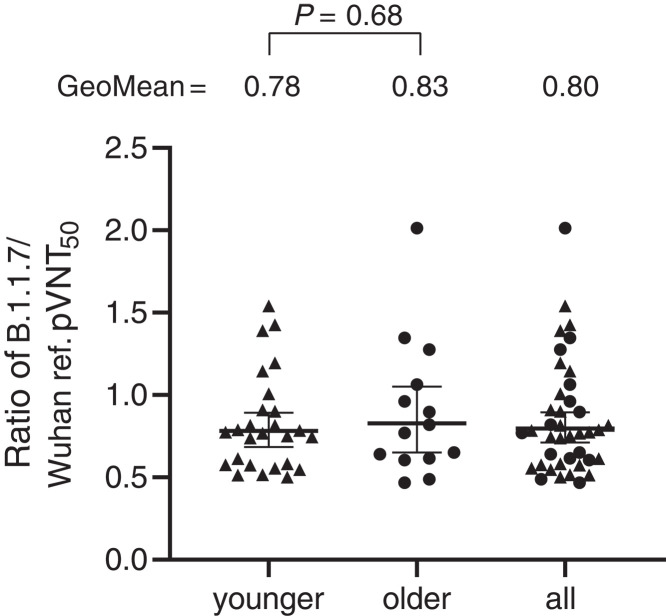
pVNT_50_ ratio of SARS-CoV-2 lineage B.1.1.7 to Wuhan reference strain spike–pseudotyped VSV. Triangles represent sera from younger adults (aged 23 to 55 years), and circles represent sera from older adults (aged 57 to 73 years). Sera were drawn on either day 29 or day 43 (7 or 21 days after vaccine dose two). Geometric means of the pVNT_50_ ratios of SARS-CoV-2 lineage B.1.1.7 to Wuhan spike–pseudotyped VSV and 95% CIs are indicated. The difference in distribution of titer ratios between younger and older adults was tested for statistical significance with a two-tailed Mann-Whitney *U* test.

To answer this question, we generated vesicular stomatitis virus (VSV)–SARS-CoV-2-S pseudoviruses bearing the Wuhan reference strain or the lineage B.1.1.7 spike protein (fig. S1). An unbiased set of sera of 40 participants in the previously reported German phase 1/2 trial (*7*)—drawn from 26 younger (aged 23 to 55 years) and 14 older adults (aged 57 to 73 years) at 7 or 21 days after the booster immunization with 30 μg of BNT162b2 (fig. S2)—was tested for neutralization of SARS-CoV-2 Wuhan and lineage B.1.1.7 spike–pseudotyped VSV by a 50% neutralization assay [50% pseudovirus neutralization titer (pVNT_50_)]. The 50% neutralization geometric mean titers (GMTs) of the sera against the SARS-CoV-2 lineage B.1.1.7 spike–pseudotyped VSV for the younger adult group and the full analysis set were slightly but statistically significantly reduced compared with the GMTs against the Wuhan reference spike–pseudotyped VSV (Fig. 1 and table S1). GMTs were not significantly different for the older adult group. The calculated geometric mean ratio with 95% confidence interval (CI) of the B.1.1.7 pseudotype and the Wuhan pseudotype GMTs was 0.78 (95% CI: 0.68 to 0.89) for the younger group and 0.83 (95% CI: 0.65 to 1.1) for the older adults [0.80 (95% CI: 0.71 to 0.89) in aggregate] (Fig. 2). No statistical difference in the ratio was observed between the younger and the older vaccinated participants.

On the basis of experience from studying antibody correlates of disease protection for influenza virus vaccines, a 20% reduced titer does not indicate a biologically relevant change in neutralization activity (*8*, *9*). The largely preserved neutralization of pseudoviruses bearing the B.1.1.7 spike by BNT162b2-immune sera makes it unlikely that the U.K. variant virus will escape BNT162b2-mediated protection.

A potential limitation of the work may be the use of a nonreplicating pseudovirus system. However, previous reports have shown good concordance between pseudotype neutralization and SARS-CoV-2 neutralization assays (*10*, *11*). Still, concordance may vary between different SARS-CoV-2 strains and remains to be demonstrated for the SARS-CoV-2 B.1.1.7 lineage. Additional experiments will be needed to confirm efficient neutralization of B.1.1.7 lineage clinical isolates. This study has evaluated sera elicited by the recommended regimen of two doses administered 21 days apart and does not provide insight into neutralization if the recommended dosing regimen is not followed. The ongoing evolution of SARS-CoV-2 necessitates continuous monitoring of the biological relevance of changes for maintained protection by the currently authorized vaccines. Unlike the protocol for influenza vaccines, the degree of reduction in neutralization that might indicate a need for a strain change has not yet been established for COVID-19 vaccines. A previous study demonstrated that BNT162b2 elicits both a polyepitopic CD8+ T cell response to the encoded spike protein and virus-neutralizing antibodies (*7*). Given the multiple potential mediators of protection elicited by BNT162b2, it is possible that vaccine efficacy could be preserved in the longer term, even with substantial losses of neutralization by vaccine-elicited sera. This view is further supported by the rapid onset of disease protection ~12 days after the first dose of BNT162b2, at a time when neutralizing antibody titers are still very low (*1*). Without an established correlate of protection, clinical effectiveness data will be needed to provide definitive assessment of vaccine-mediated protection against viral variants.

Although sustained neutralization of the current B.1.1.7 variant is reassuring, preparation for potential COVID-19 vaccine strain change is prudent. Adaptation of the vaccine to a new virus strain would be facilitated by the flexibility of mRNA-based vaccine technology.

## Supplementary Materials

science.sciencemag.org/content/371/6534/1152/suppl/DC1 Materials and Methods Figs. S1 to S3 Tables S1 and S2 Reference (*12*) MDAR Reproducibility Checklist

## Appendix

View/request a protocol for this paper from *Bio-protocol*.
